# Topical Administration of Mometasone Is Not Helpful in Post-COVID-19 Olfactory Dysfunction

**DOI:** 10.3390/life12101483

**Published:** 2022-09-24

**Authors:** Constantin A. Hintschich, Melanie Dietz, Antje Haehner, Thomas Hummel

**Affiliations:** 1 Smell & Taste Clinic, Department of Otorhinolaryngology, TU Dresden, 01307 Dresden, Germany; 2 Department of Otorhinolaryngology, Regensburg University Hospital, 93053 Regensburg, Germany

**Keywords:** COVID-19, smell, olfaction, mometasone, olfactory training

## Abstract

Persistent olfactory dysfunction is a major concern post-COVID-19, affecting up to 5% of all patients. Different therapeutic options, including mometasone nasal spray, have been recommended, only some of which have been validated for post-COVID-19 olfactory dysfunction. In this study we psychophysically assessed the effect of intranasally applied mometasone furoate on the recovery of olfaction. The spray was applied with a long applicator so that the olfactory cleft could be reached effectively. After olfactory dysfunction had been confirmed psychophysically using Sniffin’ Sticks, patients were randomly assigned to two different treatment arms: the study group (*n* = 40) underwent olfactory training and intranasal administration of mometasone furoate twice daily, whereas the control group (*n* = 46) performed olfactory training only. After a study duration of three months, psychophysical testing of olfaction was repeated using Sniffin’ Sticks. We found no benefit of an additional topical administration of mometasone furoate compared to olfactory training alone. These results psychophysically confirm two previous studies which were based on patients’ subjective self-ratings. Our findings are in contrast to current recommendations for the management of olfactory dysfunction post-COVID-19, which might have to be adapted accordingly.

## 1. Introduction

Even though a lower prevalence of acute olfactory dysfunction has been described for recent COVID-19 variants compared to the delta-variant of SARS-CoV2 [1,2], persisting hyposmia in post-COVID-19 remains a major concern. According to a recent meta-analysis, 5% of previous COVID-19 patients exhibit long-term hyposmia, although the data underlying this analysis were based on patients’ self-assessment alone [3]. Studies using psychophysical assessment of olfaction found even higher prevalence of lasting smell impairment after COVID-19 [4,5].

As hyposmia is associated with a lower quality of life, depression, sexual dysfunction, or an increased rate of accidents, any effort should be taken to recover the sense of smell [6,7,8]. Based on pre-COVID-19 studies, various treatment regimes have been recommended [9,10]: olfactory training [11], topical administration of vitamin A [12], omega-3 supplementation [13], and topical [14] and systemic corticosteroids [15]. A positive effect of some of these treatments was possibly recently confirmed for COVID-19-associated smell loss: two studies found a positive effect of olfactory training [16,17]. Hernandez et al. showed that omega-3 supplementation improves the olfactory threshold [18]. Le Bon et al. reported an increased recovery of olfaction when oral corticosteroids were administered additionally to olfactory training [19].

However, up to now, there is no evidence on the effectiveness of topical corticosteroids in the treatment of post-COVID-19 olfactory dysfunction [20,21]. Hence, the following study analyzed the effect of mometasone furoate nasal spray on the recovery of olfaction post-COVID-19.

## 2. Materials and Methods

This prospective longitudinal case control study was conducted after previous evaluation through the Institutional Review Board of the Technische Universität Dresden, Germany. The study was performed in accordance with the Declaration of Helsinki and its later amendments. After patients were given information about the course of the study and possible risks, their consent was obtained in written form.

### 2.1. Patients

Patients older than 18 years and with a persisting olfactory dysfunction after COVID-19 were recruited for this study. Exclusion criteria were known olfactory dysfunction before COVID-19, neurologic or psychiatric conditions, a previous surgery or radiotherapy of the head and neck region, a history of head trauma, chronic rhinosinusitis, and allergic rhinitis. After nasal endoscopy, olfactory function was psychophysically determined using the Sniffin’ Sticks test (Burghart Messtechnik, Wedel, Germany). Patients were then randomly assigned to the two treatment arms: the study group underwent olfactory training and topical administration of mometasone furoate (one spray per nostril twice daily) using a long applicator to reach the olfactory cleft, whereas the control group only performed olfactory training. The treatment was conducted for three months, after which the olfactory function was reassessed using the same psychophysical smell testing.

### 2.2. Olfactory Testing

Olfactory function was psychophysically assessed in the first and the follow-up visit using the Sniffin’ Sticks test [22]. The testing procedure is described in detail elsewhere [23]. In short, patients undergo three different 16-item olfactory subtests for each threshold (T), discrimination (D), and identification (I). The scores of the three subtests sum up to the composite TDI-score, the maximum score of which is 48. A score of 16 or less has been established as functional anosmia and a score of less than 30.75 as hyposmia [24].

### 2.3. Olfactory Training

In their first visit all patients were instructed how to perform olfactory training. They were given four glass jars with the following four odorants: phenyl ethyl alcohol (rose), eucalyptol (eucalyptus), citronellal (lemon), and eugenol (clove). According to an established protocol, they were asked to sniff each odorant for 30 s in the morning and the evening while fully concentrate on the smell [11].

### 2.4. Topical Administration of Mometasone Furoate

Patients of the study group intranasally administered mometasone furoate nasal spray, as established previously, for different conditions of the internal nose and paranasal sinuses (100 μg in each nostril twice daily, upright head position). The long applicator bypassed the nasal valve and hence ensured an effective drug delivery into the olfactory cleft as previously described [25,26].

### 2.5. Statistical Analysis

Data were analyzed using SPSS Statistics software (version 26, IBM, Armonk, NY, USA). Graphs were illustrated using online software Raincloud-shiny (https://gabrifc.shinyapps.io/raincloudplots/, accessed on 28 August 2022). Values are expressed as mean ± standard deviation (SD), and *p* < 0.05 was considered statistically significant. Continuous data were tested for statistical significance using unpaired two-tailed Student’s *t*-tests. Categorical data were compared between groups using Fisher’s exact test. Multivariate analysis was performed with MANOVA followed by Wilks–Lambda analysis.

## 3. Results

A total of 112 patients were enrolled in this study. During their first visit, patients were psychophysically tested for their olfactory function and randomly assigned to two different groups. Both the study group and the control group were instructed how to perform daily olfactory training. The study group additionally received a momentane nasal spray with a long applicator for daily self-administration (100 μg in each nostril twice daily). The treatment lasted three months after which patients revisited the clinic for the follow-up examination. Due to dropouts, a further SARS-CoV-2 infection, or incompliance in the application of the study protocol, a total of 26 patients had to be excluded. Hence, data of 86 patients (46.9 years ± 13.3) was analyzed. Fifty-four of the included patients were female (63%) and thirty-two were male (38%). The study group was slightly smaller than the control group (40 vs. 46 patients).

In the pretherapeutic psychophysical assessment, no significant difference in the TDI-score or its three subscores could be seen between both groups (TDI: 23.1 ± 7.8 vs. 24.6 ± 7.6; Table 1). Similarly, subjective ratings of the olfactory function did not differ significantly between the study and control group (VAS: 2.5 ± 2.2 vs. 3.4 ± 2.6; Table 1).

**Figure 1:**
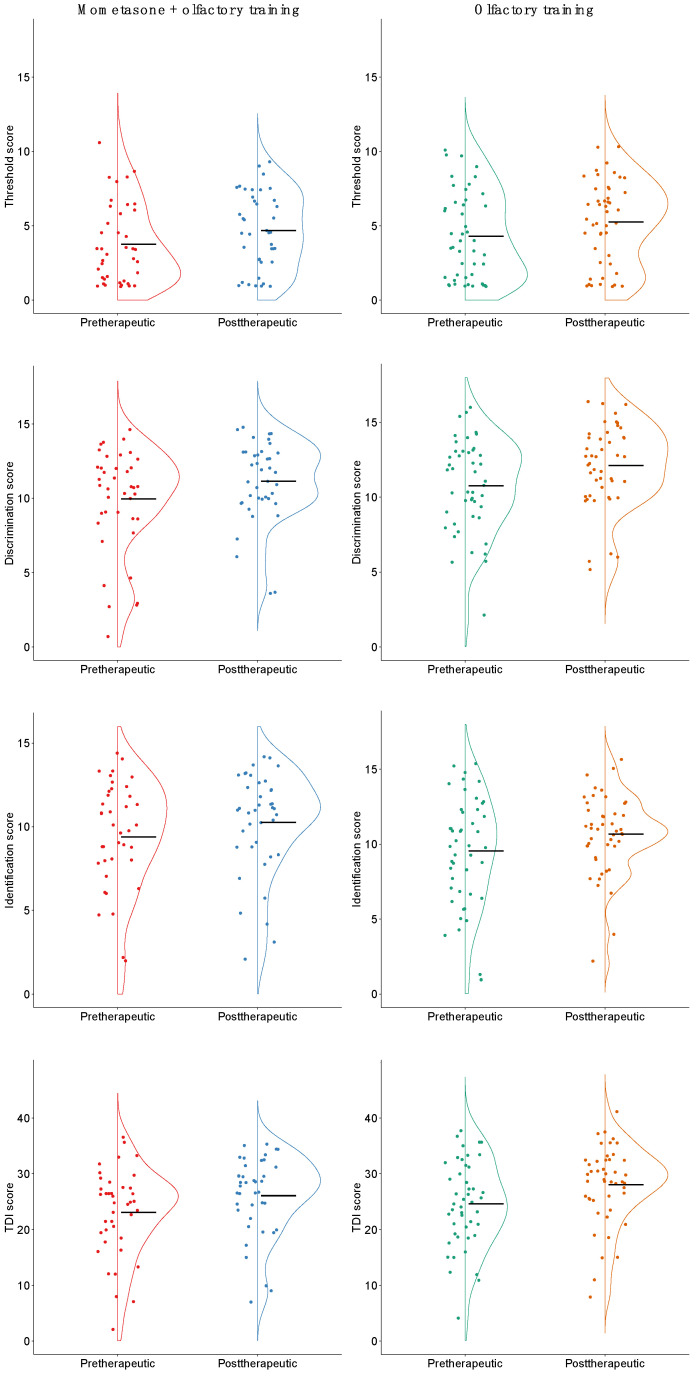
Effect of mometasone furoate and olfactory training vs. olfactory training alone on TDI score and its subscores for threshold (T), discrimination (D), and identification (I); note that intergroup differences are statistically not significant (black bar: mean).

After three months of treatment the follow-up examination was conducted on average 3.9 months after the first visit. Both groups significantly improved in TDI and all its subscores (Figure 1). However, there was no significant difference in the change in TDI and its subscores between patients who received mometasone additionally to olfactory training and those who did not. A one-way MANOVA found no statistically significant differences between the change of the three subscores of TDI on the combined dependent variables (F(3, 82) = 0.11, *p* = 0.91, partial η² = 0.32, Wilk’s Λ = 0.996). When a minimum clinical important difference (MCID) of 5.5 was considered [23] 10 patients of the study group (25%) and 15 patients of the control group (33%) improved in olfaction. Neither were of any statistical significance.

In both groups VAS ratings improved significantly. Again, no significant intergroup difference was found (ΔVAS smell: 2.6 ± 2.2 vs. 2.3 ± 2.0).

## 4. Discussion

This is the first case control study to psychophysically assess the effect of intranasally administered mometasone furoate in chronic olfactory dysfunction after COVID-19. After three months of therapy, there was no significant improvement of psychophysically assessed olfactory function when mometasone furoate was additionally administered intranasally compared to sole olfactory training.

This is consistent with two previous publications [20,21]. In these trials the authors found no benefit of an additional topical administration of mometasone furoate compared to olfactory training alone. However, the therapy duration was shorter, and olfaction was not assessed psychophysically but only by patients’ subjective self-assessment. Additionally, a recent meta-analysis identified no statistically significant difference in the olfactory recovery between patients receiving topical corticosteroids and controls [27].

In contrast, Hosseinpoor et al. showed a beneficial effect of topically administered mometasone furoate after four weeks of application [28]: in patients with subacute olfactory dysfunction due to COVID-19, olfaction recovered significantly more in the study compared to the placebo control group when assessed both subjectively and psychophysically using the UPSIT.

Even if a positive effect of systemic steroids has been shown for the treatment of pre-COVID-19 hyposmia [15,29,30], up to now there is no evidence that mometasone furoate nasal spray enhances the regeneration of olfaction in non-CRS-hyposmia [15,31].

It has to be emphasized that in the current study mometasone nasal spray was administered with a long applicator. Using such a device the spray allows a higher drug delivery to the olfactory cleft compared to conventional nasal sprays which are applied at the level of the naris [25,26,32,33]. As a consequence, application of steroid nasal sprays with an adequate applicator are much more effective in terms of a therapeutic effect compared to conventional spray, although they also may produce a minor effect through accumulation of mometasone furoate in the olfactory cleft.

In contrast to conventional nasal sprays, nasal irrigation delivers a higher drug concentration to the olfactory cleft [34]. This might, at least, explain in part why budesonide irrigation has been reported to improve olfaction in long-lasting hyposmia in post-COVID-19 (which did not include a control group) [35] and also in other etiologies [14], with olfactory loss due to (pre-COVID-19) viral infections, side effects of medication, head trauma, environmental exposure, or idiopathic causes.

However, there are some limitations to our study. Patients of the study group had olfactory dysfunction after an infection with SARS-CoV-2 for approximately two months longer than the control group. This potentially covered the effect of mometasone furoate, as the prognosis of spontaneous recovery is higher in patients with shorter history of hyposmia (control group) than in patients with a longer persistence (control group) [3]. Moreover, the follow-up interval differed slightly but significantly between both groups by approximately two weeks. However, it has to be kept in mind that the mean duration of olfactory loss was 9.5 months in the mometasone group compared to 7.2 months in the control group. Because most recovery happens during the first months following the infection it can be assumed that this difference in the duration of the olfactory loss, although statistically significant, had little clinical significance [3]. It should also be considered that otherwise the two groups appeared to be similar, especially with regard to measured olfactory function at baseline.

## 5. Conclusions

In this case control study, the topical administration of mometasone furoate did not show a beneficial effect on the recovery in patients with long-lasting olfactory dysfunction after COVID-19. Our results psychophysically confirm two previous studies which were based on patients’ self-ratings, known to be biased in many ways [36,37]. The corresponding findings are in contrast to current recommendations for the management of olfactory dysfunction in post-COVID-19, which might have to be readjusted accordingly.

## Figures and Tables

**Table 1:** Demographics, clinical information, and details of olfactory assessment before and after three months of therapy (n.s.: not significant).

	Mometasone + Olfactory Training (*n* = 40)	Olfactory Training (*n* = 46)	Significance Level
Female	27 (67.5%)	27 (58.7%)	n.s.
Age (years)	48.4 ± 11.3	45.7 ± 14.9	n.s.
Duration of hyposmia (months)	9.5 ± 2.3	7.2 ± 3.0	*p* < 0.001
Duration until follow-up (months)	4.1 ± 0.9	3.7 ± 0.8	*p* = 0.04
Pretherapeutic threshold (T)	3.7 ± 2.6	4.3 ± 2.9	n.s.
Pretherapeutic discrimination (D)	10.0 ± 3.4	10.8 ± 3.0	n.s.
Pretherapeutic identification (I)	9.4 ± 3.4	9.5 ± 3.6	n.s.
Pretherapeutic TDI	23.1 ± 7.8	24.6 ± 7.6	n.s.
Δ threshold (T)	0.9 ± 2.3	1.0 ± 2.3	n.s.
Δ discrimination (D)	1.2 ± 3.4	1.3 ± 3.0	n.s.
Δ identification (I)	0.9 ± 2.1	1.1 ± 2.3	n.s.
Δ TDI	3.0 ± 4.8	3.4 ± 5.0	n.s.
Pretherapeutic VAS smell	2.5 ± 2.2	3.4 ± 2.6	n.s.
Δ VAS smell	2.6 ± 2.2	2.3 ± 2.0	n.s.

## Data Availability

The data presented in this study are available on request from the corresponding author. The data are not publicly available due to data privacy.
